# Synthesis and Biological Evaluation of 3-Substituted-indolin-2-one Derivatives Containing Chloropyrrole Moieties

**DOI:** 10.3390/molecules16119368

**Published:** 2011-11-08

**Authors:** Yun-Zhou Jin, Da-Xu Fu, Nan Ma, Zhan-Cheng Li, Quan-Hai Liu, Lin Xiao, Rong-Hua Zhang

**Affiliations:** 1 Department of Chemistry, Tongji University, Shanghai, 200092, China; 2 Department of Pharmacology, Shanghai Institute of Pharmaceutical Industry, Shanghai, 200434, China

**Keywords:** antitumor activities, indolin-2-one, chloropyrrole, synthesis

## Abstract

Eighteen novel 3-substituted-indolin-2-ones containing chloropyrroles were synthesized and their biological activities were evaluated. The presence of a chlorine atom on the pyrrole ring was crucial to reduce cardiotoxicity. The presence of a 2-(ethyl-amino)ethylcarbamoyl group as a substituent at the C-4′ position of the pyrrole enhanced the antitumor activities notably. IC_50_ values as low as 0.32, 0.67, 1.19 and 1.22 μM were achieved against non-small cell lung cancer (A549), oral epithelial (KB), melanoma (K111) and large cell lung cancer cell lines (NCI-H460), respectively.

## 1. Introduction

Angiogenesis, an important physiological process of new capillary blood vessel formation, is essential for the survival, growth and metastasis of tumors [1,2]. Vascular endothelial growth factor (VEGF), platelet-derived growth factor (PDGF), fibroblast growth factor (FGF) and their receptor tyrosine kinases (RTKs) play key roles in angiogenesis and vascular maintenance via the autocrine and paracrine loops. Among these growth factors, by binding to its receptor VEGF is currently the most potent and clinically relevant angiogenic factor [3,4,5]. The vascular endothelial growth factor receptor (VEGFR) belongs to the RTKs superfamily and mainly comprises fms-like tyrosine kinase-1 (FLT-1, VEGFR1), kinase domain-containing receptor (KDR, VEGFR2) and fms-like tyrosine kinase-4 (FLT4, VEGFR3), which contribute to tumor progression via mediation of tumor angiogenesis and lymphangiogenesis [6,7]. Therefore, inhibition of the RTK pathway, which results in inhibition of tumor angiogenesis, has become an important strategy for discovering new antitumor drugs [8,9,10,11].

**Figure 1:**
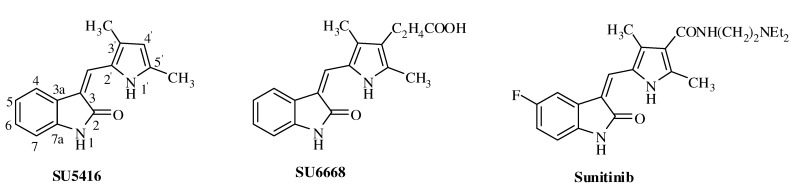
Structures of SU5416, SU6668 and sunitinib.

In recent years, indolin-2-one derivatives, especially those with heterocyclic methylene substituents attached to the C-3 position of the indolin-2-one ring, have been disclosed as potent inhibitors of RTK *in vitro* and demonstrated antiangiogenic properties *in vivo*. The (*Z*)-3-((3,5-dimethyl-1*H*-pyrrol-2-yl)methylene) indolin-2-one structure is found in many antitumor drugs [12,13]. SU5416 (Figure 1) is the first selective KDR inhibitor in clinical trials. Its derivative SU11248 (sunitinib, Figure 1) was the first RTK oral inhibitor approved by the US Food and Drug Administration (FDA) for the treatment of advanced renal cell carcinoma and gastrointestinal stromal tumors [14,15,16]. In SU6668 (Figure 1) and sunitinib, 2-carboxyethyl and 2-diethylaminoethylcarbamoyl groups were introduced into the C-4^′^ position of the pyrrole ring, respectively. Tang *et al.* reported that pyrrolo-fused-heterocycle-indolin-2-one analogues showed potent inhibition against VEGFR and good efficacies against HT-29 cell tumor xenografts in nude mice [17]. Khanwelkar *et al.* found that introduction of a ureido group into the C-6 position of 3-pyrrolemethylideneindolin-2-one enhances the potency of inhibition against VEGFR, PDGFR [18].

The introduction of halogen atoms, mainly fluorine or chlorine, has been used as a tool to enhance the potency of many pharmaceutical lead compounds [19,20]. The presence of halogen atom changes the volumetric and conformational properties, as also increases membrane permeability leading to improved absorption [21]. The importance of chlorine atoms in drug design is well documented. Besides having a larger size than fluorine, chlorine is a moderate halogen bond acceptor. Compared with bromine and iodine, the chlorine-carbon bond is stable enough to be inserted into diverse heterocycles of pharmacological value. In some drugs, subunits bearing chlorine can be accommodated in hydrophobic pockets of the biological targets [22]. Hrib *et al.* reported that the introduction of chlorine into nemonapride constituted a ubiquitous function-determining domain, while the removal of the chlorine was deleterious to the selectivity among the dopaminergic receptors [23]. Among leishmanicidal *N*-benzylcytisine derivatives, the chloro derivative demonstrated 10-fold stronger inhibition ability than others [24]. On the aromatic ring of arvanil, the replacement of a 3-methoxy group with a chlorine atom increased the capability to inhibit FAAH [25].

In previous studies, we introduced halogen atoms onto the pyrrole rings attached to an indolin-2-one framework and found that those with bromine atoms exhibited low antitumor activities, while those with chlorine atoms exhibited good antitumor activities and low cardiotoxicity. Crystallographic study of indolin-2-one with FGFR indicates that C-4′ position on the pyrrole is positioned close to the opening of the binding pocket and could be exposed to solvents [26]. We envisioned that modification of the substituted indolin-2-ones by adjusting its hydrophilicity might further enhance their biological activities. Accordingly, we introduced different N-substituents to a pyrrole-4′-formamide moiety attached to the C-3 position of indolin-2-one. We were also curious about the effects of changing the substituents on the phenyl ring on the inhibitory activities. Herein, we wish to report the synthesis of eighteen novel substituted indolin-2-ones and the *in vitro* biological evaluation of their activity against four cancer cell lines (A549, KB, K111, NCI-H460), VEGFR2 and cardiotoxicity.

## 2. Results and Discussion

### 2.1. Chemistry

**Scheme 1:**
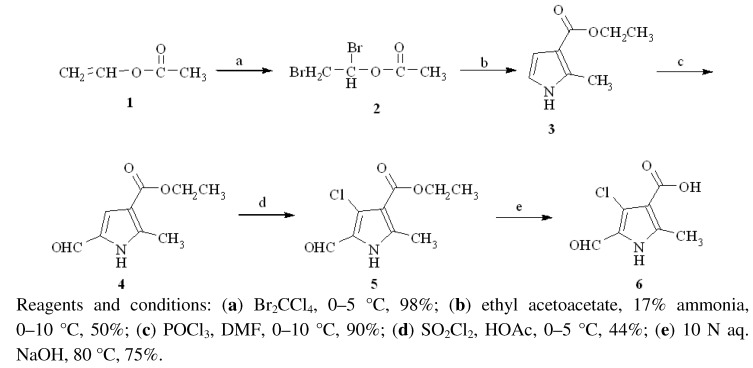
Synthesis of 4-chloro-5-formyl-2-methyl-1*H*-pyrrole-3-carboxylic acid (**6**).

4-Chloro-5-formyl-2-methyl-1*H*-pyrrole-3-carboxylic acid (**6**) was prepared from vinyl acetate (**1**) in five steps (Scheme 1). Bromination of vinyl acetate (**1**), followed by reaction with ethyl acetoacetate, afforded pyrrole derivative **3**. Treatment of **3** with freshly prepared Vilsmeier reagent gave aldehyde **4**, which was chlorinated with sulfuryl chloride to afford **5**. Compound **6** was obtained after hydrolysis of compound **5** with aqueous sodium hydroxide.

**Scheme 2:**
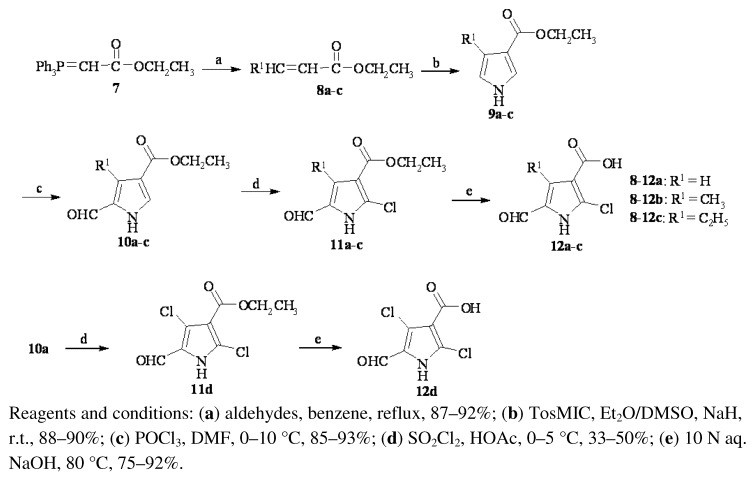
Synthesis of substituted 2-chloro-5-formyl-1*H*-pyrrole-3-carboxylic acids **12a–d**.

As shown in Scheme 2, the key intermediates **12a–d**, analogues of **6**, were prepared from ylide **7** in five steps. Wittig reaction of ylide **7** with aldehydes produced 3-alkyl/*H*-acrylates **8a–c**. Ethyl 4-alkyl/*H*-pyrrole-3-caboxylates **9a–c** were prepared by the cyclization of **8a–c** with tosylmethyl isocyanide (TosMIC). Formylation of **9a–c** afforded **10a–c**. Chlorination of substituted pyrrole **10–c** and hydrolysis of the produced chloro-substituted esters **11a–d** yielded the pyrrolecarboxylic acids **12a–d**.

**Scheme 3:**
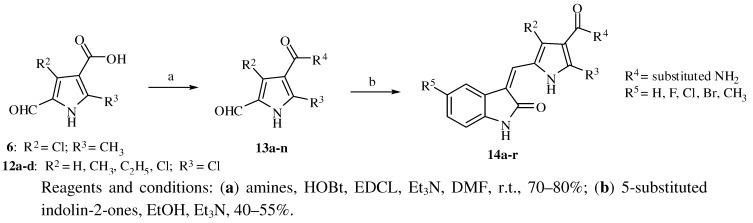
Synthesis of 3-substituted-indolin-2-one derivatives **14a–r**.

The synthetic route to target compounds **14a–r** is shown in Scheme 3 (the substituents of these compounds are listed in Table 1). Compounds **6** and **12a–d** were transformed into amides **13a–n** by the reaction with different amines. Knoevenagel condensations of **13a–n** with 5-substituted indolin-2-ones produced the target compounds **14a–r**.

### 2.2. Biological Evaluation

The antitumor activities of **14a-r** were evaluated *in vitro* on four tumor cell lines, including non-small cell lung cancer (A549), oral epithelial (KB), melanoma (K111) and large cell lung cancer cell lines (NCI-H460) by the MTT assay, and the results are summarized in Table 1.

**Table 1:** Structures of compounds **14a–r** and their antitumor activities on four tumor cell lines.

Compd.	R^2^	R^3^	R^4^	R^5^	IC_50_ ± SD (μM)			
					A549	KB	K111	NCI-H460
**14a**	Cl	CH_3_	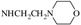	F	1.47 ± 0.13	52.91 ± 7.93	>100	>100
**14b**	Cl	CH_3_	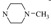	F	>100	>100	>100	>100
**14c**	Cl	CH_3_	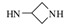	F	2.43 ± 0.26	1.35 ± 0.12	3.41 ± 0.52	1.41 ± 0.17
**14d**	Cl	CH_3_	NH(CH_2_)_2_CH_3_	F	>100	>100	>100	>100
**14e**	Cl	CH_3_	NH(CH_2_)_3_N(CH_3_)_2_	F	2.33 ± 0.31	2.88 ± 0.33	>100	>100
**14f**	Cl	CH_3_	NH(CH_2_)_2_N(CH_3_)_2_	F	1.69 ± 0.17	1.56 ± 0.08	1.35 ± 0.28	1.67 ± 0.18
**14g**	Cl	CH_3_	NH(CH_2_)_2_NHC_2_H_5_	F	1.03 ± 0.09	0.67 ± 0.08	1.19 ± 0.21	1.41 ± 0.09
**14h**	Cl	CH_3_	NH(CH_2_)_2_N(C_2_H_5_)_2_	F	1.87 ± 0.26	1.25 ± 0.10	1.79 ± 0.12	32.1 ± 3.83
**14i**	Cl	CH_3_	NH(CH_2_)_2_N(C_2_H_5_)_2_	Cl	0.32 ± 0.03	1.15 ± 0.20	>100	>100
**14j**	Cl	CH_3_	NH(CH_2_)_2_N(C_2_H_5_)_2_	Br	>100	34.21 ± 2.96	>100	>100
**14k**	Cl	CH_3_	NH(CH_2_)_2_N(C_2_H_5_)_2_	H	>100	5.09 ± 1.01	>100	>100
**14l**	Cl	CH_3_	NH(CH_2_)_2_N(C_2_H_5_)_2_	CH_3_	>100	64.42 ± 7.51	>100	>100
**14m**	Cl	Cl	NH(CH_2_)_2_N(C_2_H_5_)_2_	F	>100	>100	>100	>100
**14n**	H	Cl	NH(CH_2_)_2_N(C_2_H_5_)_2_	F	3.37 ± 0.27	4.23 ± 0.65	>100	>100
**14o**	CH_3_	Cl	NH(CH_2_)_2_N(C_2_H_5_)_2_	F	2.14 ± 0.26	1.51 ± 0.11	>100	>100
**14p**	CH_3_	Cl	NH(CH_2_)_2_NHC_2_H_5_	F	1.41 ± 0.09	0.69 ± 0.07	1.24 ± 0.16	1.66 ± 0.23
**14q**	C_2_H_5_	Cl	NH(CH_2_)_2_N(C_2_H_5_)_2_	F	16.71 ± 2.7	34.39 ± 2.90	>100	>100
**14r**	C_2_H_5_	Cl	NH(CH_2_)_2_NHC_2_H_5_	F	1.49 ± 0.11	1.43 ± 0.21	3.72 ± 0.46	1.22 ± 0.10
**Sunitinib**	CH_3_	CH_3_	NH(CH_2_)_2_N(C_2_H_5_)_2_	F	2.93 ± 0.25	2.60 ± 0.18	3.83 ± 0.43	4.79 ± 0.62
**control**	Br	CH_3_	NH(CH_2_)_2_N(C_2_H_5_)_2_	F	>100	83.39 ± 9.76	>100	>100

First, compound **14a** with a morpholine ring was examined. It was found to be effective towards A549, but ineffective on KB, K111 and NCI-H460. Compound **14b**, a nitrogen analogue of **14a**, and *N*-propyl amide **14d** had IC_50_s above 100 μM towards all four cell lines. However, the use of hydrophilic N-substituents as the basic side chain resulted in increased antitumor activities of the amides. The IC_50_s were on the μM order of magnitude. Pleasantly, **14c**, **14f**, **14g** and **14h** were effective on the four different cell lines. It should be noticed that the IC_50_ values of **14g** with a 2-(ethylamino)ethylcarbamoyl group was just 1/4–1/2 of those of sunitinib, indicating the effectiveness of changing N-substituents. By comparison with *N*,*N*-(2-(diethylamino)ethyl)amides, *N*-(2-(ethylamino)-ethyl)amides were much more active against the four cell lines, in particular against K111 and NCI-H460 (**14g** *vs.* **14h**, **14o** *vs.* **14p**, **14q** *vs.* **14r**). On the basis of the crystallographic structure study [24], the functional group at C-4′ position of pyrrole should be exposed to water. Perhaps the secondary amine structure and low steric hindrance of the terminal ethylamino group of *N*-(2-(ethylamino)ethyl)amides are favourable for the formation of hydrogen-bonds with water to improve the solubility. 

In order to examine the effects of chlorine atom on antitumor activities, two compounds **14h** and **14o** were synthesized by replacing one methyl group on the pyrrole ring of sunitinib with a chloro group. It was found that compounds **14h** and **14o** showed higher antitumor activities against A549 and KB than aunitinib. To improve the antitumor activities of compounds bearing chloro-substituted pyrroles, the effects of the number and position of chloro groups were studied. Antitumor agents **14h**, **14n** and **14o** bearing a monochloro-substituted pyrrole ring showed antitumor activities, while antitumor agent **14m** bearing a dichloro-substituted pyrrole ring had no effects. The position of the chloro group did not seem to affect the activities greatly (cf. **14g** *vs.* **14p**, **14h** *vs.* **14o**). To investigate the effects of other functional groups on the chloropyrrole ring, several substituents (hydrogen, methyl and ethyl) at the C-3′ position of pyrrole (compounds **14n**, **14o** and **14q**) were investigated. It was found that compound **14o** with a methyl group exhibited higher activity than the others. Bromine atom as the substituent on pyrrole control was found to show no antitumor activities. 

Next, the C-5 position of indolin-2-one was modified. Replacing the fluoro in **14h** with chloro (**14i**) led to a large increase in the potency towards A549 (from 1.87 μM to 0.32 μM). Unfortunately, **14i** showed little potency towards K111 and NCI-H460. When bromo, hydrogen or methyl groups were introduced to the C-5 position of indolin-2-one (**14j**, **14k**, **14l**), the potency decreased substantially (IC_50_s above 100 μM).

In conclusion, compounds **14c**, **14f**, **14g**, **14h**, **14i**, **14p** and **14r** were found to have potent antitumor activities, with **14g** showing 3-4-fold higher inhibitory activities against KB and K111 (IC_50_ = 0.67 μM, 1.19 μM) than sunitinib (IC_50_ = 2.60 μM, 3.83 μM), while compounds **14i** and **14r** showed much higher inhibitory activities against A549 and NCI-H460 (IC_50_ = 0.32 μM, 1.22 μM) than sunitinib (IC_50_ = 2.93 μM, 4.79 μM), respectively.

Seven compounds in the **14a–r** series with low IC_50_ values on the tumor cell lines were evaluated for their inhibition of VEGFR2 using an enzyme-linked immunosorbent assay (ELISA), and the data are summarized in Table 2. Except for compounds **14i** and **14r**, the other compounds all exhibited good inhibitory effects against VEGFR2. In the compounds **14c**, **14f**, **14g**, **14h** and **14p** bearing different *N*-pyrrole-4′-formamide substituents, **14g** and **14p** bearing *N*-2-(ethylamino)ethyl groups showed greatly improved biological activities against VEGFR2. It was found that **14h** which was synthesized by replacing one methyl group of Su11248 with chloro showed similar potency as Su11248 (sunitinib), but replacing the terminal *N*-diethyl of **14h** with one ethyl group (compound **14g**) greatly improved the inhibition. The different positions of the chloro atoms on the pyrrole ring of **14g** and **14p** affected the activities. Compound **14g** with a 3′-substituted chloropyrrole was more active than **14p** with a 5′-substituted chloropyrrole. In conclusion, compared with Su11248 (IC_50_ = 6.5 ± 3.0 nM), **14f**, **14g** and **14p** exhibited higher activities against VEGFR2. Among the three compounds, **14g** (IC_50_ = 5.0 ± 1.1 nM) showed the best potency. The data would suggest that the inhibition effects against other tyrosine kinase targets deserve to be evaluated in future studies.

**Table 2:** Inhibition activities of some 3-substituted-indolin-2-ones on VEGFR2.

Compd.	IC_50_ ± SD (nM)	Compd.	IC_50_ ± SD (nM)
**14c**	6.8 ± 1.8	**14i**	11.2 ± 2.6
**14f**	6.2 ± 0.8	**14p**	6.0 ± 1.2
**14g**	5.0 ± 1.1	**14r**	8.7 ± 1.3
**14h**	6.8 ± 0.8	**Sunitinib**	6.5 ± 3.0

**Figure 2:**
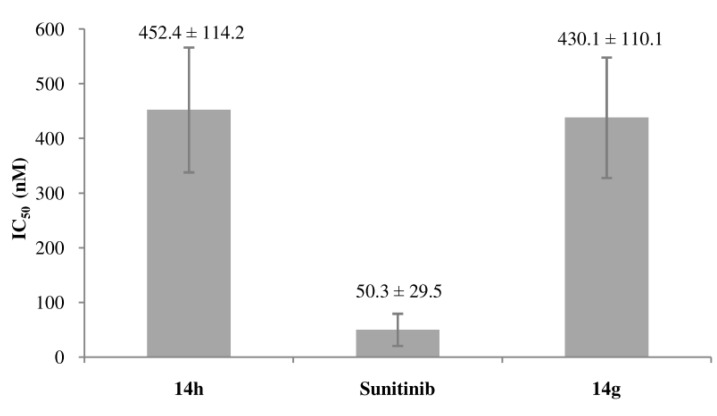
Inhibition against hERG potassium currents in HEK239 cells (IC_50_ ± SD).

Cardiotoxicity is a known serious side effect of sunitinib. The pivotal phase III metastatic renal cell carcinoma trial indicated that 21% of sunitinib-treated patients experienced a decline in left ventricular ejection fraction (LVEF) to below normal [27]. The cardiotoxicity of the synthesized compounds was examined by evaluating their inhibition against hERG potassium currents in HEK239 cells and expressed as IC_50_, as shown in Figure 2. Compound **14h**, which exhibited good *in vitro* antitumor activities and has a chloro group at the pyrrole ring C-3′ position, was selected as a candidate with low cardiotoxicity. Pleasantly, it was found that the cardiotoxicity of **14h** (IC_50_ = 452.4 nM) was about 9-fold lower than that of sunitinib (IC_50_ = 50.3 nM). The result indicated that the introduction of chlorine atom by replacing one methyl group on pyrrole ring of sunitinib with a chloro reduced the cardiotoxicity markedly. Next, compound **14g** with the best inhibition against KB, K111 and VEGFR2 was also examined and its cardiotoxicity (IC_50_ = 430.1 nM) was about 8.5-fold lower than that of sunitinib (IC_50_ = 50.3 nM). Research into the cardiotoxicity of the other compounds is in progress, and the results will be reported in the future.

## 3. Experimental

### 3.1. General

^1^H-NMR and ^13^C-NMR were recorded on a Bruker ARX-400 spectrometer with tetramethylsilane as the internal standard and CDCl_3_ or DMSO-*d_6_* as the solvent. Melting points were determined on a Kruss KSP α melting point apparatus and were uncorrected. Mass Spectra (MS) and High Resolution Mass Spectra (HRMS) were measured on a Waters Q-T Micromass spectrometer. All compounds were routinely checked by thin layer chromatography (TLC) using Huanghai silica gel HSGF-254 glass plates. Reagents (analytical grade) were obtained from commercial suppliers and used without further purification, unless otherwise noted.

### 3.2. Synthesis of 1,2-Dibromoethyl Acetate *(**2**)*

To a solution of vinyl acetate (**1**, 18.4 mL, 0.20 mol) in carbon tetrachloride (10 mL) was added bromine (13.5 mL, 0.27 mol) dropwise at 0 °C. The mixture was stirred for 1 h, and concentrated to give crude liquid product **2** which was used in the next reaction without purification. Yield: 48.4 g (98%); ^1^H-NMR (CDCl_3_): δ 2.19 (s, 3H, CH_3_), 3.85–3.95 (m, 2H, CH_2_), 6.71–6.74 (m, 1H, CH); ^13^C-NMR (CDCl_3_): δ 20.69, 33.12, 70.62, 168.03; MS (ESI): 246.86 (M+1)^+^.

### 3.3. Synthesis of Ethyl 2-Methyl-1H-pyrrole-3-carboxylate *(**3**)*

To a solution of 1,2-dibromoethyl acetate (**2**, 12.90 g, 0.15 mol) and ethyl acetoacetate (25.50 mL, 0.2 mol) was added 17% ammonia water (100 mL) dropwise at 0–10 °C during 2 h. Then the mixture was stirred at room temperature overnight. The precipitated pure **3** was collected by filtration, washed with water and dried under vacuum. Light yellow solid; Yield: 11.5 g (50%); m.p. 78.5–79.2 °C; ^1^H-NMR (CDCl_3_): δ 1.36 (t, *J* = 7.13 Hz, 3H, CH_3_), 2.54 (s, 3H, CH_3_), 4.29 (q, *J* = 7.13 Hz, 2H, CH_2_), 6.57–6.59 (m, 2H, pyrrole), 8.55 (s, 1H, NH); ^13^C-NMR (CDCl_3_): δ 13.23, 14.52, 59.40, 110.50, 111.82, 115.78, 135.25, 165.85; MS (ESI): 154.08 (M+1)^+^.

### 3.4. General Procedure for Synthesis of Ethyl 3-Substituted-acrylates ***8a–c***

A mixture of ylide **7** (10 g, 0.03 mol) and aldehydes (formaldehyde, acetaldehyde, propaldehyde) (0.03 mol) in benzene (30 mL) was refluxed for 5–6 h. Then the mixture was cooled to room temperature and filtrated. The filtrate was concentrated and purified by flash column chromatography to obtain liquid product (PE/EA = 15/1).

*Ethyl acrylate* (**8a**). Yield: 2.46 g (87%); ^1^H-NMR (CDCl_3_): δ 1.22 (t, *J =* 7.41 Hz, 3H, CH_3_), 4.15 (q, *J =* 7.41 Hz, 2H, CH_2_), 5.56–5.71 (m, 1H, CH), 6.06–6.12 (m, 1H, CH), 6.27–6.31 (m, 1H, CH); ^13^C-NMR (CDCl_3_): δ 13.82, 60.15, 127.21, 132.78, 166.80; MS (ESI): 101.10 (M+1)^+^.

*Ethyl but-2-enoate* (**8b**). Yield: 2.85 g (89%); ^1^H-NMR (CDCl_3_): δ 1.25 (t, *J =* 7.33 Hz, 3H, CH_3_), 2.01–2.06 (m, 3H, CH_3_), 4.25 (q, *J =* 7.33 Hz, 2H, CH_2_), 5.83–5.88 (m, 1H, CH), 6.78–6.84 (m, 1H, CH); ^13^C-NMR (CDCl_3_): δ 14.22, 18.25, 61.41, 143.23, 146.81, 166.52; MS (ESI): 115.12 (M+1)^+^.

*Ethyl pent-2-enoate* (**8c**). Yield: 3.31 g (92%); ^1^H-NMR (CDCl_3_): δ 1.04 (t, *J =* 7.44 Hz, 3H, CH_3_), 1.26 (t, *J =* 7.44 Hz, 3H, CH_3_), 2.16–2.24 (m, 2H, CH_2_), 4.16 (q, *J =* 7.44 Hz, 2H, CH_2_), 5.76–5.81 (m, 1H, CH), 6.96–7.03 (m, 1H, CH); ^13^C-NMR (CDCl_3_): δ 12.10, 14.21, 25.21, 60.02, 120.38, 150.50, 166.74; MS (ESI): 129.15 (M+1)^+^.

### 3.5. General Procedure for Synthesis of Ethyl 4-Alkyl/H-1H-pyrrole-3-carboxylates ***9a–c***

A suspension of TosMIC (10.5 g, 0.053 mol) and ethyl 3-substituted-acrylates **8a–c** (0.056 mol) in dry ethyl ether/DMSO (100 mL/50 mL) was added dropwise under argon to a suspension of NaH (3.75 g, 0.094 mol) in ethyl ether (50 mL) at room temperature. Then the mixture was stirred for 4–5 h. Ice water (200 mL) was added into the mixture. The aqueous phase was extracted with ethyl ether (3 × 100 mL). The organic phase was dried with anhydrous Na_2_SO_4_, concentrated under vacuum to get light brown liquid compounds **9a-c**.

*Ethyl 1H-pyrrole-3-carboxylate* (**9a**). Yield: 6.5 g (88%); ^1^H-NMR (CDCl_3_): δ 1.36 (t, *J* = 7.14 Hz, 3H, CH_3_), 4.31 (q, *J* = 7.14 Hz, 2H, CH_2_), 6.65–6.67 (m, 1H, CH), 6.76–6.79 (m, 1H, CH), 7.45–7.46 (m, 1H, CH), 9.86 (s, 1H, NH); ^13^C-NMR (CDCl_3_): δ 14.43, 59.97, 109.46, 115.98, 119.28, 123.97, 166.11; MS (ESI): 140.07 (M+1)^+^.

*Ethyl 4-methyl-1H-pyrrole-3-carboxylate* (**9b**). Yield: 7.3 g (90%); ^1^H-NMR (CDCl_3_): δ 1.28 (t, *J* = 7.12 Hz, 3H, CH_3_), 2.30 (s, 3H, CH_3_), 4.21 (q, *J* = 7.12 Hz, 2H, CH_2_), 6.54–6.57 (m, 1H, CH), 7.37–7.39 (m, 1H, CH), 9.01 (s, 1H, NH); ^13^C-NMR (CDCl_3_): δ 12.66, 14.39, 50.78, 114.28, 117.54, 120.93, 124.61. 166.45; MS (ESI): 154.06 (M+1)^+^.

*Ethyl 4-ethyl-1H-pyrrole-3-carboxylate* (**9c**). Yield: 7.9 g (89%); ^1^H-NMR (CDCl_3_): δ 1.21 (t, *J* = 7.43 Hz, 3H, CH_3_), 1.34 (t, *J* = 7.43 Hz, 3H, CH_3_), 2.76 (q, *J* = 7.43 Hz, 2H, CH_2_), 4.27 (q, *J* = 7.43 Hz, 2H, CH_2_), 6.54–6.56 (m, 1H, CH), 7.38–7.40 (m, 1H, CH), 8.65 (s, 1H, NH); ^13^C-NMR (CDCl_3_): δ 14.47, 14.59, 19.52, 59.38, 114.12, 116.14, 124.64, 128.05, 165.68; MS (ESI): 168.08 (M+1)^+^.

### 3.6. General Procedure for Synthesis of Ethyl 5-Formyl-2/4-alkyl-1H-pyrrole-3-carboxylates ***4**,**10a–c***

The Vilsmeier reagent was prepared by treatment of dry DMF (4.5 mL, 0.057 mol) with POCl_3_ (5.4 mL, 0.057 mol) at 0 °C and stirred for another hour at room temperature. In a flask, a solution of ethyl 2/4-alkyl-1*H*-pyrrole-3-carboxylates **3**,**9a–c** (0.039 mol) in DMF (10 mL) was treated with the freshly prepared Vilsmeier reagent at 0 °C. The resulting mixture was stirred for another hour at room temperature, then poured into ice (50 g), adjusted the pH to 7–8 with 10 N aqueous NaOH. The resulting mixture was heated to 60 °C for two h. The precipitated light yellow solid compounds **4**,**10a–c** were collected by filtration and dried under vacuum.

*Ethyl 5-formyl-2-methyl-1H-pyrrole-3-carboxylate* (**4**). Light yellow solid; Yield: 6.4 g (90%); m.p. 132.5–133.3 °C; ^1^H-NMR (CDCl_3_): δ 1.38 (t, *J* = 7.13 Hz, 3H, CH_3_), 2.66 (s, 3H, CH_3_), 4.32 (q, *J* = 7.13 Hz, 2H, CH_2_), 7.40–7.41 (m, 1H, CH), 9.42 (s, 1H, CHO), 10.99 (s, 1H, NH); ^13^C-NMR (CDCl_3_): δ 13.55, 14.41, 60.02, 115.49, 124.47, 130.44, 144.01, 164.30, 179.17; MS (ESI): 182.13 (M+1)^+^.

*Ethyl 5-formyl-1H-pyrrole-3-carboxylate* (**10a**). Light yellow solid; Yield: 5.6 g (85%); m.p. 84.1–85.1 °C; ^1^H-NMR (CDCl_3_): δ 1.29 (t, *J* = 7.13 Hz, 3H, CH_3_), 4.25 (q, *J* = 7.13 Hz, 2H, CH_2_), 7.34–7.35 (m, 1H, CH), 7.66–7.67 (m, 1H, CH), 9.49 (s, 1H, CHO), 10.42 (s, 1H, NH); ^13^C-NMR (CDCl_3_): δ 14.37, 60.39, 119.18, 121.62, 129.97, 133.05, 163.63, 180.04; MS (ESI): 168.07 (M+1)^+^.

*Ethyl 5-formyl-4-methyl-1H-pyrrole-3-carboxylate* (**10b**). Light yellow solid; Yield: 6.6 g (93%); m.p. 123.5–125.2 °C; ^1^H-NMR (CDCl_3_): δ 1.25 (t, *J* = 7.15 Hz, 3H, CH_3_), 2.63 (s, 3H, CH_3_), 4.23 (q, *J* = 7.15 Hz, 2H, CH_2_), 7.66–7.67 (m, 1H, CH), 9.73 (s, 1H, CHO), 10.05 (s, 1H, NH); ^13^C-NMR (CDCl_3_): δ 9.88, 11.23, 51.15, 117.11, 130.13, 130.54, 133.88, 164.51, 178.36; MS (ESI): 182.09 (M+1)^+^.

*Ethyl 4-ethyl-5-formyl-1H-pyrrole-3-carboxylate* (**10c**). Light yellow solid; Yield: 6.6 g (86%); m.p. 84.5–86.0 °C; ^1^H-NMR (CDCl_3_): δ 1.22 (t, *J* = 7.47 Hz, 3H, CH_3_), 1.29 (t, *J* = 7.47 Hz, 3H, CH_3_), 3.01 (q, *J* = 7.47 Hz, 2H, CH_2_), 4.24 (q, *J* = 7.47 Hz, 2H, CH_2_), 7.56–7.57 (m, 1H, CH), 9.64 (s, 1H, CHO), 10.32 (s, 1H, NH); ^13^C-NMR (CDCl_3_): δ 14.36, 16.92, 17.59, 59.96, 116.58, 129.86, 130.36, 140.59, 163.83, 178.35; MS (ESI): 196.15 (M+1)^+^.

### 3.7. General Procedure for Synthesis of Ethyl Chloro-substituted-5-formyl-1H-pyrrole-3-carboxylates ***5**, **11a–d***

Sulfuryl chloride (1.66 mL, 0.025 mol, for the synthesis of **11a–c**, **5**, 4.00 mL, 0.060 mol, for synthesis of **11d**) was added dropwise within 10–15 min to the solution of ethyl 2/4-alkyl-5-formy-1*H*-pyrrole-3-carboxylates **4**, **10a–c** (0.020 mol) dissolved in acetic acid (20 mL) cooled with an ice bath. After addition, the mixture was stirred for 30–60 min at room temperature, and poured into ice water (100 mL). The precipitated products **5**, **11a–d** were collected by filtration, dried under vacuum, purified by flash column chromatography (PE/EA = 5/1).

*Ethyl 4-chloro-5-formyl-2-methyl-1H-pyrrole-3-carboxylate* (**5**). Light orange solid; Yield: 1.9 g (44%); m.p. 189.3–190.0 °C; ^1^H-NMR (CDCl_3_): δ 1.36 (t, *J* = 7.13 Hz, 3H, CH_3_), 2.65 (s, 3H, CH_3_), 4.30 (q, *J* = 7.13 Hz, 2H, CH_2_), 9.45 (s, 1H, CHO), 10.09 (s, 1H, NH); ^13^C-NMR (CDCl_3_): δ 12.55, 14.41, 59.02, 116.32, 124.87, 129.78, 143.01, 162.33, 179.08; MS (ESI): 216.04 (M+1)^+^.

*Ethyl 2-chloro-5-formyl-1H-pyrrole-3-carboxylate* (**11a**). Light yellow solid; Yield: 1.8 g (45%); m.p. 123.5–125.9 °C; ^1^H-NMR (CDCl_3_): δ 1.40 (t, *J* = 7.13 Hz, 3H, CH_3_), 4.36 (q, *J* = 7.13 Hz, 2H, CH_2_), 7.40–7.41 (m, 1H, CH), 9.48 (s, 1H, CHO), 9.96 (s, 1H, NH); ^13^C-NMR (CDCl_3_): δ 14.31, 60.70, 114.79, 122.26, 122.31, 130.40, 162.13, 178.67; MS (ESI): 202.09 (M+1)^+^.

*Ethyl 2-chloro-5-formyl-4-methyl-1H-pyrrole-3-carboxylate* (**11b**). Light yellow solid; Yield: 2.2 g (50%); m.p. 173.5–174.3 °C; ^1^H-NMR (CDCl_3_): δ 1.22 (t, *J* = 7.13 Hz, 3H, CH_3_), 2.50 (s, 3H, CH_3_), 4.22 (q, *J* = 7.13 Hz, 2H, CH_2_), 9.58 (s, 1H, CHO), 9.90 (s, 1H, NH); ^13^C-NMR (CDCl_3_): δ 10.61, 11.25, 51.37, 113.35, 128.46, 129.17, 134.85, 163.36, 177.15; MS (ESI): 216.04 (M+1)^+^.

*Ethyl 2-chloro-4-ethyl-5-formyl-1H-pyrrole-3-carboxylate* (**11c**). Light yellow solid; Yield: 1.9 g (43%); m.p. 109.5–111.8 °C; ^1^H-NMR (CDCl_3_): δ 1.29 (t, *J* = 7.47 Hz, 3H, CH_3_), 1.40 (t, *J* = 7.47 Hz, 3H, CH_3_), 3.07 (q, *J* = 7.47 Hz, 2H, CH_2_), 4.37 (q, *J* = 7.47 Hz, 2H, CH_2_), 9.66 (s, 1H, CHO), 10.51 (s, 1H, NH); ^13^C-NMR (CDCl_3_): δ 14.22, 16.89, 18.11, 60.39, 112.67, 127.72, 129.59, 141.85, 162.73, 177.28; MS (ESI): 230.06 (M+1)^+^.

*Ethyl 2,4-dichloro-5-formyl-1H-pyrrole-3-carboxylate* (**11d**). Light yellow solid; Yield: 1.6 g (33%); m.p. 173.5–174.9 °C; ^1^H-NMR (CDCl_3_): δ 1.26 (t, *J* = 7.14 Hz, 3H, CH_3_), 4.30 (q, *J* = 7.14 Hz, 2H, CH_2_), 9.65 (s, 1H, CHO), 9.99 (s, 1H, NH); ^13^C-NMR (CDCl_3_): δ 13.67, 58.35, 114.78, 122.36, 124.67, 129.38, 165.14, 177.29; MS (ESI): 237.03 (M+1)^+^.

### 3.8. General Procedure for Synthesis of Chloro-substituted-5-formyl-1H-pyrrole-3-carboxylic acids ***6**, **12a–d***

Ethyl chloro-substituted-5-formyl-1*H*-pyrrole-3-carboxylates **5**, **11a–d** (0.014 mol) were dissolved in a solution of NaOH (2.80 g, 0.070 mol) in MeOH/H_2_O (2 mL/30 mL), and the mixture was refluxed for two hours, then extracted with dichloromethane (20 mL × 2). The aqueous layer was acidified with 18% hydrochloric acid to pH = 2–3 and the precipitated pure crystals **6**, **12a–d** were collected by filtration, washed with water, and dried under vacuum.

*4-Chloro-5-formyl-2-methyl-1H-pyrrole-3-carboxylic acid* (**6**). Light yellow solid; Yield: 2.0 g (75%); m.p. > 290 °C; ^1^H-NMR (DMSO-*d_6_*): δ 2.45 (s, 3H, CH_3_), 9.59 (s, 1H, CHO), 12.48 (s, 1H, NH), 13.21 (s, 1H, COOH); ^13^C-NMR (DMSO-*d_6_*): δ 14.25, 112.21, 127.11, 131.12, 143.03, 164.34, 177.54; MS (ESI): 188.06 (M+1)^+^.

*2-Chloro-5-formyl-1H-pyrrole-3-carboxylic acid* (**12a**). Light yellow solid; Yield: 19.4 g (80%); m.p. > 290 °C; ^1^H-NMR (DMSO-*d_6_*): δ 7.38–7.40 (m, 1H, CH), 9.45 (s, 1H, CHO), 12.67 (s, 1H, NH), 13.51 (s, 1H, COOH); ^13^C-NMR (DMSO-*d_6_*): δ 114.24, 122.88, 127.89, 131.48, 163.66, 180.11; MS (ESI): 174.01 (M+1)^+^.

*2-Chloro-5-formyl-4-methyl-1H-pyrrole-3-carboxylic acid* (**12b**). Light yellow solid; Yield: 2.4 g (92%); m.p. > 290 °C; ^1^H-NMR (DMSO-*d_6_*): δ 2.49 (s, 3H, CH_3_), 9.66 (s, 1H, CHO), 12.64 (s, 1H, NH), 13.21 (s, 1H, COOH); ^13^C-NMR (DMSO-*d_6_*): δ 10.90, 113.06, 128.09, 129.19, 133.76, 164.42, 178.80; MS (ESI): 188.04 (M+1)^+^.

*2-Chloro-4-ethyl-5-formyl-1H-pyrrole-3-carboxylic acid* (**12c**). Light yellow solid; Yield: 2.5 g (89%); m.p. 248.3–250.2 °C; ^1^H-NMR (DMSO-*d_6_*): δ 1.14 (t, *J* = 7.28 Hz, 3H, CH_3_), 2.99 (q, *J* = 7.28 Hz, 2H, CH_2_), 9.65 (s, 1H, CHO), 12.63 (s, 1H, NH), 13.20 (s, 1H, COOH); ^13^C-NMR (DMSO-*d_6_*): δ 17.24, 17.82, 112.22, 128.07, 128.42, 140.35, 164.25, 178.70; MS (ESI): 202.10 (M+1)^+^.

*2,4-Dichloro-5-formyl-1H-pyrrole-3-carboxylic acid* (**12d**). Light yellow solid; Yield: 2.4 g (81%); m.p. > 290 °C; ^1^H-NMR (DMSO-*d_6_*): δ 9.76 (s, 1H, CHO), 12.32 (s, 1H, NH), 13.15 (s, 1H, COOH); ^13^C-NMR (DMSO-*d_6_*): δ 114.28, 122.75, 124.37, 128.93, 165.09, 178.07; MS (ESI): 209.01 (M+1)^+^.

### 3.9. General Procedure for Synthesis of 3-Substituted-indolin-2-ones ***14a–r***

To a solution of compounds **6**, **12a–d** (0.006 mol) in DMF (10 mL) were added amines (0.0072 mol), EDC·HCl (1.73 g, 0.009 mol), HOBt (1.22 g, 0.009 mol) and Et_3_N (1.22 g, 0.012 mol). The mixture was stirred for 20 h at room temperature and poured into water (20 mL). The mixture was extracted with dichloromethane (20 mL × 3). The organic phase was washed with saturated aqueous NaCl (20 mL × 2), dried with anhydrous Na_2_SO_4_, and concentrated to get the crude products **13a–n** which were used in the next step without purification. To a solution of **13a–n** dissolved in ethanol (8.0 mL) were added indolin-2-ones (0.0066 mol) and Et_3_N (0.0010 mol). The mixture was heated to reflux for 2–4 h. The precipitated crystals were collected by filtration, purified by recrystallization from ethanol or by flash column chromatography (DCM/MeOH = 15/1~12/1), and dried under vacuum at 80–90 °C to get target compounds **14a–r**.

*(Z)-4-Chloro-5-((5-fluoro-2-oxoindolin-3-ylidene)methyl)-2-methyl-N-(2-morpholinoethyl)-1H-pyrrole -3-carboxamide* (**14a**). Yellow solid; Yield: 0.8 g (32%); m.p. 268.5–269.7 °C; ^1^H-NMR (CDCl_3_): δ 2.56 (t, *J =* 7.12 Hz, 4H, CH_2_ × 2), 2.63 (t, *J =* 5.82 Hz, 2H, CH_2_), 2.71 (s, 3H, CH_3_), 3.55–3.59 (m, 2H, CH_2_), 3.77 (t, *J =* 7.12 Hz, 4H, CH_2_ × 2), 6.83–7.70 (m, 6H, aromatic, vinyl, CONH, indolin-2-one NH), 13.58 (s, 1H, pyrrole NH); ^13^C-NMR (CDCl_3_): δ 14.20, 36.30, 53.58, 57.30, 66.80, 107.32, 110.96, 117.99, 118.67, 118.82, 122.61, 124.46, 125.24, 126.53, 135.86, 137.29, 157.11, 162.50, 169.95; HRMS (ESI) *m/z* (M+H)^+^: calculated for C_21_H_23_ClFN_4_O_3_^+^: 433.1443, found 433.1440.

*(Z)-3-((3-Chloro-5-methyl-4-(4-methylpiperazine-1-carbonyl)-1H-pyrrol-2-yl)methylene)-5-fluoroindolin-2-one* (**14b**)*.* Yellow solid; Yield: 0.8 g (32%); m.p. 276.7–278.5 °C; ^1^H-NMR (CDCl_3_): δ 2.36 (s, 3H, CH_3_), 2.45 (s, 3H, CH_3_), 2.50 (t, *J =* 7.12 Hz, 4H, CH_2_ × 2), 3.10 (t, *J =* 7.12 Hz, 4H, CH_2_ × 2), 6.82–7.41 (m, 4H, aromatic, vinyl, CONH), 7.99 (s, 1H, indolin-2-one NH), 13.48 (s, 1H, pyrrole NH); ^13^C-NMR (CDCl_3_): δ 13.22, 46.08, 47.26, 54.88, 105.97, 110.20, 113.76, 116.87, 118.13, 119.85, 122.58, 124.48, 126.71, 133.75, 135.39, 158.02, 163.83, 169.76; HRMS (ESI) *m/z* (M+H)^+^: calculated for C_20_H_21_ClFN_4_O_2_^+^: 403.1335, found 403.1337.

*(Z)-N-(azetidin-3-yl)-4-chloro-5-((5-fluoro-2-oxoindolin-3-ylidene)methyl)-2-methyl-1H-pyrrole-3- carboxamide* (**14c**). Yellow solid; Yield: 0.7 g (33%); m.p. 230.5–232.0 °C; ^1^H-NMR (DMSO-*d_6_*): δ 1.23 (s, 1H, NH), 2.47 (s, 3H, CH_3_), 3.99–4.13 (m, 4H, CH_2_ × 2), 4.73–4.82 (m, 1H, CH), 6.84–8.26 (m, 5H, aromatic, vinyl, CONH), 10.95 (s, 1H, indolin-2-one NH), 13.75 (s, 1H, pyrrole NH); ^13^C-NMR (DMSO-*d_6_*): δ 11.02, 42.07, 53.04, 107.21, 110.30, 114.08, 117.98, 119.76, 120.01, 124.45, 126.32, 127.01, 131.43, 135.55, 158.42, 164.07, 170.12; HRMS (ESI) *m/z* (M+H)^+^: calculated for C_18_H_17_ClFN_4_O_2_^+^: 375.1368, found 375.1369.

*(Z)-4-Chloro-5-((5-fluoro-2-oxoindolin-3-ylidene)methyl)-2-methyl-N-propyl-1H-pyrrole-3-carboxamide* (**14d**). Yellow solid; Yield: 1.0 g (43%); m.p. > 290 °C; ^1^H-NMR (DMSO-*d_6_*): δ 0.92 (t, *J* = 7.32 Hz, 3H, CH_3_), 1.49–1.58 (m, 2H, CH_2_), 2.46 (s, 3H, CH_3_), 3.18–3.23 (m, 2H, CH_2_), 6.87–7.84 (m, 5H, aromatic, vinyl, CONH), 11.09 (s, 1H, indolin-2-one NH), 13.85 (s, 1H, pyrrole NH); ^13^C-NMR (DMSO-*d_6_*): δ 11.94, 13.97, 22.89, 41.04, 107.12, 110.92, 114.09, 115.67, 118.55, 118.96, 121.82, 122.65, 124.38, 135.81, 136.56, 159.35, 162.66, 169.95; HRMS (ESI) *m/z* (M+H)^+^: calculated for C_18_H_18_ClFN_3_O_2_^+^: 362.1072 (M+1), found 362.1073.

*(Z)-4-Chloro-N-(3-(dimethylamino)propyl)-5-((5-fluoro-2-oxoindolin-3-ylidene)methyl)-2-methyl-1H-pyrrole-3-carboxamide* (**14e**). Yellow solid; Yield: 1.1 g (45%); m.p. 235.1–237.3 °C; ^1^H-NMR (DMSO-*d_6_*): δ 0.87–0.91 (m, 2H, CH_2_), 2.20 (s, 6H, CH_3_× 2), 2.41 (t, *J* = 6.76 Hz, 2H, CH_2_), 2.49 (s, 3H, CH_3_), 3.31–3.35 (m, 2H, CH_2_), 6.87–7.75 (m, 5H, aromatic, vinyl, CONH), 11.10 (s, 1H, indolin-2-one NH), 13.87 (s, 1H, pyrrole NH); ^13^C-NMR (DMSO-*d_6_*): δ 13.90, 26.40, 34.37, 43.23, 56.60, 107.35 110.08, 112.33, 113.49, 118.03, 120.29, 123.80, 124.67, 125.17, 133.72, 139.49, 157.27, 164.55, 168.66; HRMS (ESI) *m/z* (M+H)^+^: calculated for C_20_H_23_ClFN_4_O_2_^+^: 405.1494, found 405.1491.

*(Z)-4-Chloro-N-(2-(dimethylamino)ethyl)-5-((5-fluoro-2-oxoindolin-3-ylidene)methyl)-2-methyl-1H- pyrrole-3-carboxamide* (**14f**). Yellow solid; Yield: 1.0 g (42%); m.p. 271.8–274.2 °C; ^1^H-NMR (DMSO-*d_6_*): δ 2.21 (s, 6H, CH_3_× 2), 2.41 (t, *J* = 6.76 Hz, 2H, CH_2_), 2.49 (s, 3H, CH_3_), 3.32 (q, *J* = 6.76 Hz, 2H, CH_2_), 6.87–7.75 (m, 5H, aromatic, vinyl, CONH), 11.09 (s, 1H, indolin-2-one NH), 13.87 (s, 1H, pyrrole NH); ^13^C-NMR (DMSO-*d_6_*): δ 13.91, 34.42, 43.25, 56.66, 110.20, 112.47, 113.33, 115.58, 118.02, 118.13, 120.50, 123.89, 125.39, 133.81, 139.53, 157.34, 164.70, 168.81; HRMS (ESI) *m/z* (M+H)^+^: calculated for C_19_H_21_ClFN_4_O_2_^+^: 391.1337, found 391.1338.

*(Z)-4-Chloro-N-(2-(ethylamino)ethyl)-5-((5-fluoro-2-oxoindolin-3-ylidene)methyl)-2-methyl-1H- pyrrole-3-carboxamide* (**14g**). Yellow solid; Yield: 0.8 g (33%); m.p. 211.0–211.8 °C; ^1^H-NMR (DMSO-*d_6_*): δ 1.04 (t, *J* = 7.13 Hz, 3H, CH_3_), 1.21 (s, 1H, NH), 2.48 (s, 3H, CH_3_), 2.61 (q, *J* = 7.13 Hz, 2H, CH_2_), 2.72 (t, *J* = 7.13 Hz, 2H, CH_2_), 3.32 (q, *J* = 7.13 Hz, 2H, CH_2_), 6.87–7.94 (m, 5H, aromatic, vinyl, CONH), 11.27 (s, 1H, indolin-2-one NH), 13.87 (s, 1H, pyrrole NH); ^13^C-NMR (DMSO-*d_6_*): δ 14.12, 15.31, 38.21, 43.51, 48.52, 107.02, 111.05, 114.15, 118.30, 118.78, 122.60, 124.43, 125.96, 126.63, 135.86, 137.11, 158.84, 162.76, 169.95; HRMS (ESI) *m/z* (M+H)^+^: calculated for C_19_H_21_ClFN_4_O_2_^+^: 391.1337, found 391.1340.

*(Z)-4-Chloro-N-(2-(diethylamino)ethyl)-5-((5-fluoro-2-oxoindolin-3-ylidene)methyl)-2-methyl-1H- pyrrole-3-carboxamide* (**14h**). Yellow solid; Yield: 1.1 g (44%); m.p. 245.6–246.9 °C; ^1^H-NMR (DMSO-*d_6_*): δ 1.19 (t, *J* = 7.12 Hz, 6H, CH_3_× 2), 2.39 (s, 3H, CH_3_), 2.69–2.77 (m, 6H, CH_2_× 3), 3.31–3.43 (m, 2H, CH_2_), 6.78–7.77 (m, 5H, aromatic, vinyl, CONH), 11.07 (s, 1H, indolin-2-one NH), 14.12 (s, 1H, pyrrole NH); ^13^C-NMR (DMSO-*d_6_*): δ 8.42, 13.83, 34.21, 48.21, 50.52, 107.26, 109.98, 112.24, 117.99, 120.05, 120.19, 123.76, 124.97, 125.24, 133.66, 139.35, 156.86, 164.52, 168.55; HRMS (ESI) *m/z* (M+H)^+^: calculated for C_21_H_25_ClFN_4_O_2_^+^: 419.1650, found 419.1653.

*(Z)-4-Chloro-5-((5-chloro-2-oxoindolin-3-ylidene)methyl)-N-(2-(diethylamino)ethyl)-2-methyl-1H- pyrrole-3-carboxamide* (**14i**). Yellow solid; Yield: 1.1 g (40%); m.p. 246.0–247.0 °C; ^1^H-NMR (DMSO-*d_6_*): δ 1.14 (t, *J* = 7.26 Hz, 6H, CH_3_× 2), 2.45 (s, 3H, CH_3_), 2.62–2.72 (m, 6H, CH_2_× 3), 3.46–3.48 (m, 2H, CH_2_), 6.67–7.65 (m, 5H, aromatic, vinyl, CONH), 11.14 (s, 1H, indolin-2-one NH), 14.19 (s, 1H, pyrrole NH); ^13^C-NMR (DMSO-*d_6_*): δ 8.51, 14.04, 34.44, 48.31, 50.73, 113.28, 117.30, 117.77, 118.27, 120.34, 123.86, 125.44, 126.45, 126.47, 126.54, 136.06, 139.58, 164.81, 168.48; HRMS (ESI) *m/z* (M+H)^+^: calculated for C_21_H_25_Cl_2_N_4_O_2_^+^: 435.1355, found 435.1354.

*(Z)-5-((5-bromo-2-oxoindolin-3-ylidene)methyl)-2-chloro-N-(2-(diethylamino)ethyl)-4-methyl-1H- pyrrole-3-carboxamide* (**14j**). Yellow solid; Yield: 1.2 g (39%); m.p. 248.1–248.5 °C; ^1^H-NMR (DMSO-*d_6_*): δ 1.01 (t, *J* = 7.10 Hz, 6H, CH_3_ × 2), 2.24 (s, 3H, CH_3_), 2.61–2.68 (m, 6H, CH_2_ × 3), 3.46–3.50 (m, 2H, CH_2_), 6.78–7.76 (m, 5H, aromatic, vinyl, CONH), 10.85 (s, 1H, indolin-2-one NH), 13.53 (s, 1H, pyrrole NH); ^13^C-NMR (DMSO-*d_6_*): δ 8.66, 13.94, 36.14, 50.31, 52.70, 113.26, 116.87, 117.27, 119.07, 121.15, 123.54, 125.24, 126.05, 126.49, 126.54, 136.06, 139.24, 162.56, 168.98; HRMS (ESI) *m/z* (M+H)^+^: calculated for C_21_H_24_BrClN_4_O_2 _ and C_21_H_26_Cl_2_N_4_O_2_^2+^: 478.0771 (M), 480.0742 (M+2), found 478.0773, 480.0743.

*(Z)-4-Chloro-N-(2-(diethylamino)ethyl)-2-methyl-5-((2-oxoindolin-3-ylidene)methyl)-1H-pyrrole-3- carboxamide* (**14k**). Yellow solid; Yield: 1.1 g (43%); m.p. 238.5–240.1 °C; ^1^H-NMR (DMSO-*d_6_*): δ 1.01 (t, *J* = 7.25 Hz, 6H, CH_3_× 2), 2.43 (s, 3H, CH_3_), 2.52–2.57 (m, 6H, CH_2_× 3), 3.32–3.36 (m, 2H, CH_2_), 6.91–7.74 (m, 6H, aromatic, vinyl, CONH), 11.10 (s, 1H, indolin-2-one NH), 13.82 (s, 1H, pyrrole NH); ^13^C-NMR (DMSO-*d_6_*): δ 8.45, 13.92, 34.37, 48.25, 50.70, 109.53, 112.17, 117.30, 118.24, 118.57, 119.42, 121.83, 124.12, 127.28, 136.62, 137.81, 138.93, 165.02, 168.83; HRMS (ESI) *m/z* (M+H)^+^: calculated for C_21_H_26_ClN_4_O_2_^+^: 401.1744, found 401.1741.

*(Z)-4-Chloro-N-(2-(diethylamino)ethyl)-2-methyl-5-((5-methyl-2-oxoindolin-3-ylidene)methyl)-1H- pyrrole-3-carboxamide* (**14l**). Yellow solid; Yield: 1.0 g (40%); m.p. 238.2–240.0 °C; ^1^H-NMR (DMSO-*d_6_*): δ 0.99 (t, *J* = 7.09 Hz, 6H, CH_3_× 2), 2.31 (s, 3H, CH_3_), 2.50 (s, 3H, CH_3_), 2.53–2.59 (m, 6H, CH_2_× 3), 3.39–3.43 (m, 2H, CH_2_), 6.79–7.64 (m, 5H, aromatic, vinyl, CONH), 10.98 (s, 1H, indolin-2-one NH), 13.82 (s, 1H, pyrrole NH); ^13^C-NMR (DMSO-*d_6_*): δ 8.46, 13.89, 20.36, 34.41, 48.29, 50.81, 109.11, 112.05, 116.91, 118.57, 118.91, 119.01, 124.15, 127.71, 131.24, 135.58, 135.76, 138.68, 165.25, 168.88; HRMS (ESI) *m/z* (M+H)^+^: calculated for C_22_H_28_ClN_4_O_2_^+^: 415.1901, found 415.1903.

*(Z)-2,4-Dichloro-N-(2-(diethylamino)ethyl)-5-((5-fluoro-2-oxoindolin-3-ylidene)methyl)-1H-pyrrole-3-carboxamide* (**14m**). Yellow solid; Yield: 1.1 g (42%); m.p. 269.1–270.3 °C; ^1^H-NMR (DMSO-*d_6_*): δ 1.03 (t, *J* = 7.17 Hz, 6H, CH_3_× 2), 2.45–2.50 (m, 6H, CH_3_× 2), 3.25–3.30 (m, 2H, CH_2_), 6.89–7.97 (m, 5H, aromatic, vinyl, CONH), 11.15 (s, 1H, indolin-2-one NH), 13.92 (s, 1H, pyrrole NH); ^13^C-NMR (DMSO-*d_6_*): δ 12.45, 35.15, 48.60, 55.12, 109.25, 112.34, 115.55, 118.03, 119.04, 120.11, 122.31, 126.36, 127.40, 128.11, 134.88, 158.57, 162.27, 169.66; HRMS (ESI) *m/z* (M+H)^+^: calculated for C_20_H_22_Cl_2_FN_4_O_2_^+^: 439.1027, found 439.1024.

*(Z)-2-Chloro-N-(2-(diethylamino)ethyl)-5-((5-fluoro-2-oxoindolin-3-ylidene)methyl)-1H-pyrrole-3- carboxamide* (**14n**). Yellow solid; Yield: 1.0 g (39%); m.p. 233.5–234.7 °C; ^1^H-NMR (DMSO-*d_6_*): δ 0.98 (t, *J* = 7.16 Hz, 6H, 2 × CH_3_), 2.51–2.56 (m, 6H, 3 × CH_2_), 3.25–3.30 (m, 2H, CH_2_), 6.88–8.00 (m, 6H, aromatic, vinyl, CONH, pyrrole), 11.17 (s, 1H, indolin-2-one NH), 14.32 (s, 1H, pyrrole NH); ^13^C-NMR (DMSO-*d_6_*): δ 12.37, 37.40, 47.21, 51.92, 107.15, 111.25, 114.55, 118.53, 119.64, 120.02, 121.39, 126.50, 127.37, 128.11, 135.96, 158.78, 161.77, 169.98; HRMS (ESI) *m/z* (M+H)^+^: calculated for C_20_H_23_ClFN_4_O_2_^+^: 405.1494, found 405.1497.

*(Z)-2-Chloro-N-(2-(diethylamino)ethyl)-5-((5-fluoro-2-oxoindolin-3-ylidene)methyl)-4-methyl-1H- pyrrole-3-carboxamide* (**14o**). Yellow solid; Yield: 1.0 g (41%); m.p. 243.4–244.0 °C; ^1^H-NMR (DMSO-*d_6_*): δ 0.99 (t, *J* = 7.05 Hz, 6H, CH_3_× 2), 2.44 (s, 3H, CH_3_), 2.60–2.70 (m, 6H, CH_2_× 3), 3.51–3.55 (m, 2H, CH_2_), 6.87–7.86 (m, 5H, aromatic, vinyl, CONH), 11.15 (s, 1H, indolin-2-one NH), 14.53 (s, 1H, pyrrole NH); ^13^C-NMR (DMSO-*d_6_*): δ 11.01, 12.36, 37.55, 47.02, 51.92, 107.16, 110.92, 113.92, 117.92, 119.64, 119.75, 125.11, 126.73, 127.01, 130.49, 135.42, 158.82, 162.68, 170.22; HRMS (ESI) *m/z* (M+H)^+^: calculated for C_21_H_25_ClFN_4_O_2_^+^: 419.1650 , found 419.1652.

*(Z)-2-Chloro-N-(2-(ethylamino)ethyl)-5-((5-fluoro-2-oxoindolin-3-ylidene)methyl)-4-methyl-1H- pyrrole-3-carboxamide* (**14p**). Yellow solid; Yield: 0.8 g (34%); m.p. 215.0–216.8 °C; ^1^H-NMR (DMSO-*d_6_*): δ 1.03 (t, *J* = 7.13 Hz, 3H, CH_3_), 1.23 (s, 1H, NH), 2.43 (s, 3H, CH_3_), 2.60 (q, *J* = 7.13 Hz, 2H, CH_2_), 2.70 (t, *J* = 7.13 Hz, 2H, CH_2_), 3.32(q, *J* = 7.13 Hz, 2H, CH_2_), 6.87–7.89 (m, 5H, aromatic, vinyl, CONH), 11.27 (s, 1H, indolin-2-one NH), 14.50 (s, 1H, pyrrole NH); ^13^C-NMR (DMSO-*d_6_*): δ 10.97, 14.36, 38.37, 43.30, 48.06, 107.31, 110.96, 113.98, 118.01, 119.62, 119.90, 125.11, 126.76, 127.08, 130.45, 135.46, 157.67, 163.06, 170.23; HRMS (ESI) *m/z* (M+H)^+^: calculated for C_19_H_21_ClFN_4_O_2_^+^: 391.1337, found 391.1335.

*(Z)-2-Chloro-N-(2-(diethylamino)ethyl)-4-ethyl-5-((5-fluoro-2-oxoindolin-3-ylidene)methyl)-1H-pyrrole-3-carboxamide* (**14q**). Yellow solid; Yield: 1.2 g (45%); m.p. 245.2–246.8 °C; ^1^H-NMR (DMSO-*d_6_*): δ 0.98 (t, *J* = 7.09 Hz, 6H, CH_3_× 2), 1.12 (t, *J* = 7.09 Hz, 3H, CH_3_), 2.44–2.62 (m, 6H, CH_2_× 3), 2.92 (q, *J* = 7.09 Hz, 2H, CH_2_), 3.29–3.35 (m, 2H, CH_2_), 6.88–7.89 (m, 5H, aromatic, vinyl, CONH), 11.14 (s, 1H, indolin-2-one NH), 14.51 (s, 1H, pyrrole NH); ^13^C-NMR (DMSO-*d_6_*): δ 12.31, 17.29, 17.97, 37.53, 46.99, 51.93, 107.27, 110.93, 113.93, 118.08, 124.85, 125.93, 126.96, 127.06, 135.42, 137.26, 158.84, 162.82, 170.19; HRMS (ESI) *m/z* (M+H)^+^: calculated for C_22_H_27_ClFN_4_O_2_^+^: 433.1807, found 433.1809.

*(Z)-2-Chloro-4-ethyl-N-(2-(ethylamino)ethyl)-5-((5-fluoro-2-oxoindolin-3-ylidene)methyl)-1H-pyrrole-3-carboxamide* (**14r**). Yellow solid; Yield: 0.9 g (36%); m.p. 232.0–233.1 °C; ^1^H-NMR (DMSO-*d_6_*): δ 1.07–1.14 (m, 6H, CH_3_× 2), 1.22 (s, 1H, NH), 2.71 (q, *J* = 7.15 Hz, 2H, CH_2_), 2.80 (t, *J* = 7.15 Hz, 2H, CH_2_), 2.93 (q, *J* = 7.15 Hz, 2H, CH_2_), 3.44 (q, *J* = 7.15 Hz, 2H, CH_2_), 6.88–8.10 (m, 5H, aromatic, vinyl, CONH), 11.20 (s, 1H, indolin-2-one NH), 14.58 (s, 1H, pyrrole NH); ^13^C-NMR (DMSO-*d_6_*): δ 13.75, 17.26, 17.98, 37.87, 43.08, 47.57, 107.29, 110.98, 113.96, 118.19, 119.07, 119.57, 124.83, 125.96, 126.98, 135.48, 137.23, 158.84, 163.23, 170.18; HRMS (ESI) *m/z* (M+H)^+^: calculated for C_20_H_23_ClFN_4_O_2_^+^: 405.1494, found 405.1497.

### 3.10. Cell Culture

Non-small cell lung cancer (A549), oral epithelial (KB), large cell lung cancer (NCI-H460) and melanoma cell lines (K111) were cultured in Dulbecco’s modified Eagle medium (GIBCO) containing 10% (v/v) inactivated newborn calf serum (Boren Bio-Pharmaceutical Co., Ltd.: Guangzhou, China), 1 nM L-glutamine (SANGON), 105 units/mL penicillin, 100 µg/mL streptomycin. Cells were grown in a 5% CO_2_ incubator at 37 °C.

### 3.11. Cell Growth Inhibition Assay

The antitumor activities were determined by the MTT assay. Cells (2 × 10^3^ cells/well) were seeded in 96-well plates. After incubation overnight, each compound, which was dissolved in dimethylsulfoxide (DMSO) and diluted with phosphate buffer solution (PBS), was added to each well and the cells were cultured for another two days at 37 °C. 20 μL (5 mg/mL) MTT solution was added to per well and the culture continued for another 3–4 h. The medium was removed and 100 μL DMSO was added to dissolve formazan crystals. Absorbance was measured at 492 nm using an ELISA reader (Thermo). The antitumor activities were expressed as IC_50_.

### 3.12. VEGFR2 Inhibition Assay

The VEGFR2 inhibition assay was performed by enzyme-linked immunosorbent assay (ELISA) in 96-well plates pre-coated with 2.5 µg/well Poly (Glu, Tyr)_4:1_ as a substrate. Each well was treated with 90 µL of 5.0 µM ATP (Amresco) solution and 10 µL of one of the seven compounds at varying concentrations, which were diluted in reaction buffer (50 mM HEPES pH 7.4, 20 mM MgCl_2_, 0.1 mM Na_3_VO_4_, 1 mM DTT). The known VEGFR2 inhibitor, Su11248 (sunitinib), was used as a positive control, and 0.1% (v/v) DMSO was utilized as the negative control. The reaction was initiated by adding 1 µL/well of VEGFR2 tyrosine kinase. After incubation for 1 h at 37 °C, the plates were washed three times with phosphate buffered saline containing 0.1% Tween-20 (T-PBS). Next, anti-phosphotyrosine (PY99; 1:1,000 dilution, 100 μL/well) antibody was added. After 1 h incubation at room temperature, the plates were washed three times, and goat anti-mouse IgG horseradish peroxidase (100 μL/well of 1:2,000 dilution) diluted in T-PBS was added. The plates were reincubated at room temperature for 1 h, and washed as before. Finally, color development solution (0.03% H_2_O_2_ and 2 mg/mL *o*-phenylenediamine in 0.1 M citrate buffer, pH 5.5, 100 µL/well) was added and the plates were incubated at room temperature until color emerged. The reaction was terminated by the addition of 2 M H_2_SO_4_ (50 µL/well), and A_492_ was measured using a tunable wavelength microplate reader (Molecular Devices SPECTRAMAX190). The inhibition rate was calculated using the equation: [1 − (A_492_/A_492 control_)] × 100%.

## 4. Conclusions

In summary, we have prepared eighteen novel 3-substituted-indolin-2-one derivatives containing different N-substituted-chloropyrroloformamides, and evaluated their biological activities. The antitumor activities were affected by the N-substituents in the amides. Compounds **14c**, **14f**, **14g**, **14h**, **14i**, **14p** and **14r** exhibited potent antitumor activities against the four tested tumor cell lines. Compound **14g** showed the best inhibition against VEGFR2. Compounds **14g** and **14h**, with a chloro group attached to the pyrrole moiety, showed low cardiotoxicity. Further experiments for the eighteen derivatives would be required for evaluating the inhibition activities against other RTKs VEGFR3, PDGFRβ and FGFR1 to conclude a comprehensive structure-activity relationship.
